# Associations in Cigarette Smoking and Health Conditions by Race/Ethnicity Among a Diverse Sample of Patients Receiving Treatment in a Federally Qualified Health Care Setting in Chicago

**DOI:** 10.1089/heq.2022.0056

**Published:** 2023-02-02

**Authors:** Larisa A. Burke, Alana D. Steffen, Sandeep Kataria, Karriem S. Watson, Robert A. Winn, Damilola Oyaluade, Barbara Williams, Cherdsak Duangchan, Carl Asche, Alicia K. Matthews

**Affiliations:** ^1^ College of Nursing, University of Illinois Chicago, Chicago, Illinois, USA.; ^2^ Oncology Bioinformatics, University of Illinois Cancer Center, Chicago, Illinois, USA.; ^3^ Office of Director, All of Us Research Program, National Institute of Health, Bethesda, Maryland, USA.; ^4^ VCU Massey Cancer Center, Richmond, Virginia, USA.; ^5^ University of Illinois College of Medicine, Peoria, Illinois, USA.

**Keywords:** tobacco use, smoking cessation, health status disparities, community health centers, medical records, ethnic groups

## Abstract

**Purpose:**

To examine the association of cigarette use and smoking-related health conditions by race/ethnicity among diverse and low-income patients at a federally qualified health center (FQHC).

**Methods:**

Demographics, smoking status, health conditions, death, and health service use were extracted from electronic medical data for patients seen between September 1, 2018, and August 31, 2020 (*n*=51,670). Smoking categories included everyday/heavy smoker, someday/light smoker, former smoker, or never smoker.

**Results:**

Current and former smoking rates were 20.1% and 15.2%, respectively. Males, Black, White, non-partnered, older, and Medicaid/Medicare patients were more likely to smoke. Compared with never smokers, former and heavy smokers had higher odds for all health conditions except respiratory failure, and light smokers had higher odds of asthma, chronic obstructive pulmonary disease, emphysema, and peripheral vascular disease. All smoking categories had more emergency department visits and hospitalizations than never smokers. The associations between smoking status and health conditions differed by race/ethnicity. White patients who smoked had a greater increase in odds of stroke and other cardiovascular diseases compared with Hispanic and Black patients. Black patients who smoked had a greater increase in odds of emphysema and respiratory failure compared with Hispanic patients. Black and Hispanic patients who smoked had a greater increase in emergency care use compared with White patients.

**Conclusion:**

Smoking was associated with disease burden and emergency care and differed by race/ethnicity.

**Health Equity Implications:**

Resources to document smoking status and offer cessation services should be increased in FQHCs to promote health equity for lower income populations.

## Introduction

In recent years, cigarette smoking has declined within the United States; however, many Americans continue to smoke.^1^ As of 2020, 12.5% of U.S. adults reported being current cigarette users.^2^ More than 16 million Americans live with serious smoking-related illnesses, such as cancer, heart disease, stroke, chronic obstructive pulmonary disease (COPD), emphysema, lung disease, chronic bronchitis, and other related health conditions.^1^ Studies have shown that individuals who smoke for longer periods tend to have fair or poor health outcomes and progressively higher mortality risk.^3^ In addition, significant disparities in cigarette use exist across groups defined by race, ethnicity, educational level, and socioeconomic status and across regions of the country.^1,4^

Despite significant progress stemming from tobacco prevention and treatment programs, current tobacco use remains elevated among individuals from lower socioeconomic backgrounds.^5^ In 2015, smoking prevalence among persons living below the poverty level (26.1%) was nearly double that of persons living at or above the poverty level (13.9%).^6^ More recent data demonstrated that youth at or below the federal poverty line had a two times higher rate of smoking onset (17%) than youth above the poverty margin (8%).^7^ Lower household income has also been correlated with higher nicotine dependency and a lower likelihood of cessation.^8^

Federally qualified health centers (FQHCs) provide health care to many individuals from lower socioeconomic groups. In 2012, 21 million patients, primarily uninsured (36%) or publicly insured (49%), received care at an FQHCs.^9^ FQHCs are safety net clinics that serve low-income and uninsured patients. A recent study found that the state's overall proportion of tobacco use in FHQCs was 25.8% compared with 20.6% in the general population.^10^ FQHCs can serve as points of intervention for smoking cessation among at-risk-lower-income people, given the high rates of tobacco burden observed in these clinical practice settings.

However, recent research on safety net clinics reveals that disparities exist in those who are at risk to smoke and the implementation of smoking cessation best practices. Demographic factors such as racial group, sex, age, insurance status, and chronic health conditions have been associated with risk of smoking, the documenting of smoking status and receipt of cessation services in safety net clinics.^11–14^ In addition, although racial health disparities in the United States are large and pervasive,^15,16^ research is limited on racial disparities in chronic health conditions and in the associations between race/ethnicity, smoking, and health conditions in FQHC populations.

The current study objectives are to examine factors associated with tobacco use and associated health conditions among current patients of a large urban-serving FQHC system. Specific objectives are to: (1) determine demographic factors associated with smoking, (2) examine associations between smoking and health conditions, (3) examine racial differences in health conditions, and (4) examine racial differences in associations between smoking and health conditions among FQHC patients. We hypothesize that there will be significant differences in the demographics of patients by smoking status, health conditions will be more prevalent for patients with any history of smoking, and that the rates of health conditions and the associations between smoking and health conditions will differ for Black, Hispanic, and White patients.

## Methods

This study utilized a cross-sectional descriptive study design. Data were from patients receiving care at Mile Square Health Center (MSHC). MSHC is a network of six community-located FQHCs co-owned and operated by the University of Illinois Hospital and Health Sciences System. De-identified electronic health record data were pulled for all patients with an FQHC clinic visit between September 1, 2018, and August 31, 2020 (*n*=51,670), the 2 years preceding the hospital system's transition to a new electronic medical record system. Patients may have had data from a single visit to multiple visits covering <1 month to 10.7 years. The institutional review board of the University of Illinois at Chicago approved the study (IRB No. 2020-1621).

### Measures

#### Smoking status

Current smoking status at the time of the data pull included: current daily smoker, current someday smoker, smoker-current status unknown, former smoker, never smoker, unknown if ever smoked, heavy tobacco smoker, light tobacco smoker, and missing.

#### Health status

Any presence in the patient record across visits of an *International Classification of Diseases*-10 code for 10 health conditions linked to or exacerbated by smoking was determined including: asthma, COPD, lung cancer, emphysema, respiratory failure, diabetes, hypertension, stroke, peripheral vascular disease, and other cardiovascular diseases. Mortality, as documented in the health record, was also extracted.

Health service utilization data included aggregate counts of visits to the FQHC clinic and/or the affiliated hospital system. The data included the number of FQHC clinic visits, emergency department (ED) visits, number of hospitalizations, and the number of days hospitalized.

Demographic variables included age, race, ethnicity, primary language, gender, relationship status, insurance type, body mass index (BMI), area of residence as determined by zip code, and months as a patient. Racial categories included American Indian or Alaska Native, Asian, Native Hawaiian or other Pacific Islander, Black or African American, White, Hispanic, and Other. Ethnicity included Hispanic or Latino.

### Analysis

STATA v.17 was used for data analysis. Data were restricted to patients whose current smoking status was known and who resided in FQHC service areas as determined by patient residential zip codes. Of the full sample (*n*=51,670), 47,205 (91.4%) patients had smoking status and 38,842 (75.2%) patients lived within clinic service areas resulting in an analytic sample of 35,807 (69.2%). Smoking status was collapsed into four categories: everyday/heavy smoker, someday/light smoker, former smoker, and never smoker because heavy and light smoking categories were sparsely populated. Race and ethnicity were merged so that any patient with Hispanic or Latino ethnicity was categorized as Hispanic regardless of race.

Demographic differences in smoking status were tested using chi-square tests for categorical demographic factors and Kruskal–Wallis tests for continuous factors (Table 1). To test the relationship between smoking status, health conditions and mortality logistic regression models were run adjusted for age, sex, race/ethnicity, insurance, relationship status, BMI, residence area, and months as a patient (Table 2). Zero-inflated Poisson models with an offset for the duration of time as a patient were run for the number of ED visits, hospitalizations, and the number of days hospitalized.

**Table 1.** Demographic, Clinical Characteristics, Health Conditions, and Service Use by Smoking Status in the Total Sample (*n*=35,807)

	Everyday/heavy smoker (*n*=4896)	Someday/light smoker (*n*=2290)	Former smoker (*n*=5230)	Never smoker (*n*=23,391)	Total (*n*=35,807)
Residence					
South Chicago	2040 (16.4)	852 (6.9)	1757 (14.1)	7787 (62.6)	12,436 (34.7)
Southwest Chicago	661 (9.6)	388 (5.6)	1023 (14.8)	4824 (70.0)	6896 (19.3)
West/Northwest Chicago	2083 (13.7)	973 (6.4)	2275 (15.0)	9830 (64.8)	15,161 (42.3)
Berwyn/Cicero	112 (8.5)	77 (5.9)	175 (13.3)	950 (72.3)	1314 (3.7)
Age, years	45 (35–58)	39 (30–53)	48 (35–62)	36 (27–50)	39 (29–54)
Gender					
Male	2211 (19.0)	1084 (9.3)	2132 (18.3)	6232 (53.5)	11,659 (32.6)
Female	2683 (11.1)	1206 (5.0)	3098 (12.8)	17,156 (71.1)	24,143 (67.4)
Missing	2 (0.0)	0 (0.0)	0 (0.0)	3 (0.0)	5 (0.0)
Race/ethnicity					
White	392 (14.5)	196 (7.3)	496 (18.3)	1620 (59.9)	2704 (7.8)
Black	3470 (17.7)	1331 (6.8)	2868 (14.6)	11,920 (60.9)	19,589 (56.4)
Asian	57 (6.3)	34 (3.7)	57 (6.3)	764 (83.8)	912 (2.6)
Hispanic	697 (6.8)	577 (5.6)	1520 (14.7)	7522 (72.9)	10,316 (29.7)
Other	151 (12.7)	83 (7.0)	145 (12.2)	809 (68.1)	1188 (3.4)
Missing	129 (2.6)	69 (3.0)	144 (2.8)	756 (3.2)	1098 (3.1)
Language					
English	4576 (14.6)	2126 (6.8)	4589 (14.6)	20,140 (64.1)	31,431 (89.3)
Spanish	187 (5.4)	116 (3.4)	556 (16.1)	2595 (75.1)	3454 (9.8)
Other	43 (13.4)	17 (5.3)	28 (8.7)	233 (72.6)	321 (0.9)
Missing	90 (1.8)	31 (1.4)	57 (1.1)	423 (1.8)	601 (1.7)
Marital					
Partnered	635 (9.1)	325 (4.7)	1157 (16.6)	4860 (69.7)	6977 (20.1)
Not partnered	4076 (14.7)	1901 (6.9)	3927 (14.2)	17,780 (64.2)	27,684 (79.9)
Missing	185 (3.8)	64 (2.8)	146 (2.8)	751 (3.2)	1146 (3.2)
Insurance					
Private	1018 (7.9)	749 (5.8)	1514 (11.8)	9588 (74.5)	15,093 (42.1)
Medicaid	2809 (18.6)	1039 (6.9)	2298 (15.2)	8947 (59.3)	3236 (9.0)
Medicare	519 (16.0)	185 (5.7)	894 (27.6)	1638 (50.6)	12,869 (35.9)
Self-pay	523 (11.7)	312 (7.0)	508 (11.4)	3121 (69.9)	4464 (12.5)
Other	16 (15.4)	2 (1.9)	10 (9.6)	76 (73.1)	104 (0.3)
Missing	11 (0.2)	3 (0.1)	6 (0.1)	21 (0.1)	15,093 (42.1)
Body mass index	28.5 (24–34)	29.4 (25–35)	30.8 (26–36)	29.7 (25–36)	29.7 (25–35)
Missing	133 (2.8)	57 (2.6)	59 (1.2)	525 (2.3)	744 (2.2)
Conditions					
Asthma	860 (17.6)	353 (15.4)	1026 (19.6)	3076 (13.1)	5315 (14.8)
COPD	405 (8.3)	127 (5.5)	452 (8.6)	449 (1.9)	1433 (4.0)
Lung cancer	22 (0.4)	3 (0.1)	54 (1.0)	25 (0.1)	104 (0.3)
Emphysema	48 (1.0)	13 (0.6)	60 (1.1)	17 (0.1)	138 (0.4)
Respiratory failure	18 (0.4)	1 (0.04)	27 (0.5)	40 (0.2)	86 (0.2)
Diabetes	910 (18.6)	356 (15.5)	1471 (28.1)	3494 (14.9)	6231 (17.4)
Hypertension	1943 (39.7)	750 (32.7)	2570 (49.1)	6667 (28.5)	11,930 (33.3)
Stroke	142 (2.9)	45 (2.0)	179 (3.4)	320 (1.4)	686 (1.9)
PVD	267 (5.4)	95 (4.1)	468 (8.9)	613 (2.6)	1443 (4.0)
Other CVD	732 (15.0)	302 (13.2)	1266 (24.2)	2826 (12.1)	5126 (14.3)
Death	24 (0.5)	9 (0.4)	52 (1.0)	68 (0.3)	153 (0.4)
Health services					
Any ED visit	1960 (40.0)	908 (40.0)	2549 (48.7)	9918 (42.4)	15,335 (42.8)
Any IP stay	1088 (22.2)	454 (19.8)	1763 (33.7)	5651 (24.2)	8956 (25.0)
Months as a patient	51 (10–95)	48 (9–95)	65 (24–110)	55 (13–105)	56 (13–1041)
Number of visits	6 (2–17)	4 (2–13)	7 (2–21)	4 (2–14)	5 (2–15)

Statistical differences between smoking groups, all *p*<0.001. Values are represented as *n* (%) for categorical variables and median (interquartile range) for continuous variables.

COPD, chronic obstructive pulmonary disease; CVD, cardiovascular disease; ED, emergency department; IP, inpatient; PVD, peripheral vascular disease.

**Table 2.** Rates of Health Conditions by Smoking Status Adjusted for Age, Sex, Race/Ethnicity, Insurance, Partner Status, Body Mass Index, Region, and Months as a Patient (*n*=33,026)

	Everyday/heavy smoker	Someday/light smoker	Former smoker	Never smoker	Everyday/heavy smoker vs. never smoker, OR (95% CI)	Someday/light smoker vs. never smoker, OR (95% CI)	Everyday/heavy smoker vs. someday/light smoker, OR (95% CI)	Former smoker vs. never smoker, OR (95% CI)
	Adjusted %							
Asthma	18.5	16.6	18.4	13.6	1.5 (1.3–1.6)^***^	1.3 (1.1–1.5)^***^	1.2 (1–1.3)	1.5 (1.3–1.6)^***^
COPD	7.7	5.9	5.7	2.3	3.9 (3.4–4.6)^***^	2.8 (2.3–3.5)^***^	1.4 (1.1–1.8)^*^	2.7 (2.4–3.2)^***^
Emphysema	0.9	0.6	0.7	0.1	2.4 (1.3–4.4)^***^	0.6 (0.1–2.7)^***^	3.9 (0.9–16.5)	4.7 (2.8–7.7)^***^
Lung cancer	0.4	0.1	0.7	0.1	10.6 (5.8–19.2)^**^	6.9 (3.2–14.8)	1.5 (0.8–2.9)	8 (4.5–14.1)^***^
Respiratory failure	0.4	0.1	0.3	0.2	1.8 (1.0–3.3)	0.3 (0–1.9)	7.1 (0.9–53.8)	1.6 (0.9–2.7)
Diabetes	18.4	17.4	19.6	17.4	1.1 (1.0–1.2)	1.0 (0.9–1.2)	1.1 (0.9–1.3)	1.2 (1.1–1.3)^***^
Hypertension	35.5	34	37.3	33.3	1.1 (1.0–1.2)^**^	1.0 (0.9–1.2)	1.1 (1–1.3)	1.3 (1.2–1.4)^***^
Stroke	2.5	2.1	2.1	1.7	1.5 (1.2–1.9)^***^	1.3 (0.9–1.8)	1.2 (0.8–1.7)	1.2 (1–1.5)^*^
PVD	5.4	4.7	5.4	3.3	1.8 (1.6–2.2)^***^	1.6 (1.2–2)^***^	1.2 (0.9–1.5)	1.8 (1.6–2.1)^***^
Other CVD	15	14.4	17.9	13.8	1.1 (1.0–1.2)^*^	1.1 (0.9–1.2)	1.1 (0.9–1.3)	1.5 (1.4–1.6)^***^
Death	0.5	0.4	0.6	0.4	1.3 (0.8–2.2)	1.1 (0.5–2.4)	1.2 (0.5–2.6)	1.6 (1.1–2.3)^*^

	Adjusted count				IRR (95% CI)	IRR (95% CI)	IRR (95% CI)	IRR (95% CI)
Number of ED	2.7	3.1	2.1	2.0	1.3 (1.3–1.4)^***^	1.6 (1.5–1.6)^***^	0.8 (0.8–0.9)^***^	1.0 (1.0–1.1)^***^
Number of IP	1.0	1.0	1.1	0.8	1.2 (1.1–1.2)^***^	1.2 (1.1–1.3)^***^	1.0 (0.9–1.0)^***^	1.2 (1.2–1.3)^***^
Days of IP	4.1	4.0	4.6	3.5	1.1 (1.1–1.1)^***^	1.2 (1.1–1.2)^***^	1.0 (0.9–1.0)^***^	1.3 (1.2–1.3)^***^

Death model *n*=32,661.

^*^ *p*<0.05; ^**^p<0.01; ^***^p<0.001.

CI, confidence interval; IRR, incidence rate ratio; OR, odds ratio.

Those who had never smoked were compared with former, current someday/light smokers, and current everyday/heavy smokers, and someday/light smokers were compared with everyday/heavy smokers. Regression models were also run to test for racial differences in health conditions (Table 3) and the racial differences in the associations between smoking status and health conditions (Table 4). For these models, smoking categories were dichotomized (ever vs. never smoked) and less populated racial groups (<5%) were excluded (American Indian or Alaska Native, Asian, Native Hawaiian or other Pacific Islander, Other) to avoid sparse data. An interaction term between race/ethnicity and smoking status was added to the models to test for racial differences when comparing ever versus never smokers.

**Table 3.** Rates of Health Conditions by Smoking Status and Race/Ethnicity Adjusted for Age, Sex, Insurance, Partner Status, Body Mass Index, Region, and Months as a Patient (*n*=31,115)

	Black	Hispanic	White	Black vs. White, OR (95% CI)	Hispanic vs. White, OR (95% CI)	Black vs. Hispanic, OR (95% CI)
	Adjusted %					
Asthma	16.6	12.5	17.7	0.9 (0.8–1.0)	0.6 (0.6–0.7)^***^	1.4 (1.3–1.6)^***^
COPD	4.6	2.6	6.6	0.6 (0.5–0.8)^***^	0.3 (0.3–0.4)^***^	1.9 (1.6–2.3)^***^
Emphysema	0.3	0.2	0.4	0.7 (0.4–1.2)	0.4 (0.2–0.8)^*^	1.8 (1.0–3.2)^*^
Lung cancer	0.4	0.2	0.6	1.0 (0.5–2.0)	0.6 (0.3–1.4)	1.6 (1.0–3.0)^*^
Respiratory failure	0.3	0.3	0.1	2.2 (0.5–9)	2.3 (0.5–10.2)	0.9 (0.5–1.7)
Diabetes	17.7	20.9	12.7	1.6 (1.4–1.9)^***^	2.1 (1.8–2.5)^***^	0.8 (0.7–0.8)^***^
Hypertension	38.6	29.3	29.8	1.8 (1.6–2.0)^***^	1.0 (0.8–1.1)	1.9 (1.7–2)^***^
Stroke	2.2	1.7	1.8	1.1 (0.8–1.6)	0.9 (0.6–1.2)	1.3 (1.0–1.7)^*^
PVD	4.4	3.8	4.9	0.9 (0.7–1.1)	0.7 (0.6–1.0)^*^	1.2 (1.0–1.4)^*^
Other CVD	15.8	13.9	14.0	1.2 (1.0–1.4)^*^	1.0 (0.8–1.1)	1.2 (1.1–1.3)^***^
Death	0.4	0.5	0.5	0.9 (0.5–1.6)	1.1 (0.6–2.1)	0.8 (0.5–1.2)

	Adjusted count			IRR (95% CI)	IRR (95% CI)	IRR (95% CI)
Number of ED	2.2	2.3	2.5	0.9 (0.8–0.9)^***^	0.9 (0.9–0.9)^***^	1.0 (1.0–1.0)
Number of IP	0.8	1.1	1.5	0.6 (0.6–0.7)^***^	0.8 (0.8–0.9)^***^	0.8 (0.7–0.8)^***^
Days of IP	3.5	4.7	5.9	0.7 (0.7–0.7)^***^	0.9 (0.8–0.9)^***^	0.8 (0.8–0.8)^***^

Death model *n*=30,781.

^*^ *p*<0.05; ^***^p<0.001.

IRR, incidence rate ratio.

**Table 4.** Rates of Health Conditions by Smoking Status and Race/Ethnicity Adjusted for Age, Sex, Insurance, Partner Status, Body Mass Index, Region, and Months as a Patient (*n*=31,115)

	Black		Hispanic		White		Change in odds/rate for smokers, Black vs. White, OR (95% CI)	Change in odds/rate for smokers, Hispanic vs. White, OR (95% CI)	Change in odds/rate for smokers, Black vs. Hispanic, OR (95% CI)
	Ever smoked	Never smoked	Ever smoked	Never smoked	Ever smoked	Never smoked			
	Adjusted %								
Asthma	19.5	15.1	15.4	11	21.8	15.4	0.9 (0.7–1.1)	1.0 (0.7–1.3)	0.9 (0.8–1.1)
COPD	7.3	2.6	3.9	1.6	10.3	3.9	1.0 (0.7–1.5)	0.8 (0.5–1.4)	1.2 (0.9–1.6)
Emphysema	0.6	0.1	0.3	0.2	0.6	0.2	1.0 (0.1–9.2)	0.1 (0–1.4)	6.8 (1.9–24)^**^
Lung cancer	0.9	0.1	0.3	0.1	1.2	0.1	1.3 (0.2–7.4)	0.5 (0.1–3.0)	2.9 (0.9–9.0)
Respiratory failure	0.4	0.2	0.2	0.4	0.1	0.1	2.4 (0.1–41.2)	0.5 (0.0–9.9)	4.9 (1.4–17.0)^*^
Diabetes	18.4	17.1	21.8	20.3	14.8	11.2	0.8 (0.6–1.0)	0.8 (0.5–1.0)	1.0 (0.9–1.2)
Hypertension	40.3	37.5	30.2	28.7	32.2	28.2	0.9 (0.7–1.1)	0.8 (0.7–1.1)	1.1 (0.9–1.2)
Stroke	2.4	2	1.9	1.5	2.9	0.9	0.3 (0.1–0.7)^**^	0.4 (0.2–0.8)^*^	0.9 (0.6–1.4)
PVD	5.6	3.4	4.6	3.2	6.7	3.4	0.8 (0.5–1.3)	0.7 (0.4–1.1)	1.2 (0.9–1.6)
Other CVD	17.1	14.9	15.2	13	17.7	11.6	0.7 (0.5–0.9)^**^	0.7 (0.5–0.9)^*^	1.0 (0.8–1.2)
Death	0.5	0.3	0.6	0.5	0.6	0.5	1.4 (0.4–4.9)	1.0 (0.3–3.7)	1.4 (0.7–2.8)

	Adjusted count						IRR (95% CI)	IRR (95% CI)	IRR (95% CI)
Number of ED	2.4	2.0	2.8	2.0	2.5	2.6	1.2 (1.1–1.3)^***^	1.4 (1.3–1.6)^***^	0.8 (0.8–0.9)^***^
Number of IP	1.0	0.7	1.3	1.1	1.3	1.7	1.8 (1.5–2.0)^***^	1.6 (1.4–1.8)^***^	1.1 (1–1.2)^***^
Days of IP	4.2	3.0	5.1	4.5	5.7	6.0	1.5 (1.4–1.6)^***^	1.2 (1.1–1.3)^***^	1.2 (1.2–1.3)^***^

Smoking status was dichotomized and patients who ever smoked (current and former smokers) were compared with patients who never smoked. Change in odds for health outcomes for ever smokers versus never smokers was compared across racial groups by interaction terms. Death model *n*=30,781.

^*^ *p*<0.05; ^**^p<0.01; ^***^p<0.001.

## Results

### Demographics and health conditions

The median number of months as a patient was 56 months, and the median number of FQHC clinic visits was 5 (Table 1). The median age was 39 years (range 18–108 years), and the majority of patients were women (67.4%). The racial background of patients included Black or African American *n*=19,589 (56.4%); Hispanic or Latino *n*=10,316 (29.7%); White non-Hispanic *n*=2704 (7.8%); Asian *n*=912 (2.6%); American Indian or Alaskan Native *n*=49 (0.1%); Native Hawaiian or other Pacific Islander *n*=21 (0.1%), and Other *n*=1118 (3.2%).

The most common respiratory condition for patients was asthma (15%). COPD was reported in 4% of patients. Diagnoses of lung cancer, emphysema, and respiratory failure occurred in less than 1% of patients. Seventeen percent of patients had a diagnosis of diabetes, and over a third of patients had a hypertension diagnosis. Stroke and peripheral vascular disease occurred in less than 5% of patients. Other cardiovascular diagnoses were present for 14% of patients. Of all patients, 43% had an ED visit within the university health system, and 25% had an inpatient stay/hospitalization. The medical record noted that 153 (0.4%) patients in the sample had died.

### Smoking status

Overall, 13.7% of patients were everyday/heavy smokers, 6.4% were someday/light smokers, 14.6% were former smokers, and 14.6% were never smokers (Table 1). The prevalence of current smoking was highest for patients residing in South, West, or Northwest Chicago, and for patients who were older, male, Black, White, English-speaking, non-partnered, or had Medicaid/Medicare (Table 1). Former smoking was more common among patients who were male, White, older, Medicare insured, had a higher BMI, or had a higher duration of time as a patient and a greater number of clinic visits.

### Associations between smoking status and health conditions

Table 2 compares health conditions for three categories of smokers (former, current someday/light, current everyday/heavy) with never smokers adjusted for age, sex, race/ethnicity, insurance, relationship status, BMI, area of residence, and the number of months as a patient. Compared with patients who never smoked, smokers had higher odds of asthma, COPD, emphysema, and peripheral vascular disease rates. In addition, everyday/heavy smokers had higher odds of COPD than someday/light smokers. Everyday/heavy smokers and former smokers had higher odds of lung cancer, hypertension, stroke, and other cardiovascular diseases than never smokers. The rates of these conditions were not increased for someday/light smokers.

Former smokers were more likely to have diabetes or to have died than never smokers. Current and former smokers had a higher number of ED visits, hospitalizations, and days of hospitalization than never smokers. Someday/light smokers had the highest number of ED visits, followed by everyday/heavy smokers and former smokers. Former smokers had the highest number of hospitalizations and days of hospitalizations.

### Racial differences in health conditions

Table 3 displays health conditions for Hispanic, non-Hispanic Black, and non-Hispanic White patients who smoked (current or former) compared with those who never smoked. Some smoking related diagnoses and health service use differed by race/ethnicity. Among conditions that differed by race, Hispanic patients had lower odds of smoking-related health conditions compared to Black and White patients, although Hispanic patients did have higher odds of diabetes. White patients had higher odds of COPD than Black and Hispanic patients. Black patients had higher odds of hypertension than White and Hispanic patients, higher odds of stroke than Hispanic patients, and higher odds of diabetes than White patients. White patients had the highest number of ED visits and hospitalizations, followed by Hispanic patients. Black patients had the lowest number of ED visits and hospitalizations. Deaths reported in the medical record did not differ by race/ethnicity.

### Racial differences in associations between smoking status and health conditions

Smoking status was dichotomized, and patients who ever smoked (current and former smokers) were compared with never smokers by racial groups using race/ethnicity by smoking interaction terms (Table 4). Black patients had a greater increase in the odds of emphysema and respiratory failure for smokers compared with never smokers than Hispanic patients who had a smaller increase in the odds of emphysema and decreased odds of respiratory failure for those who smoked. White patients had a greater increase in the odds of stroke or other cardiovascular diagnoses for smokers versus never smokers compared with Black or Hispanic patients. Hispanic patients had the greatest increase in the number of ED visits for smokers versus never smokers and Black patient had the greatest increase in the number and days of hospitalization. White smokers did not have an increased number of ED visits or hospitalization days, and the number of hospitalizations was significantly less compared with White nonsmokers.

## Discussion

Twenty percent of our patient population were current smokers. Demographic characteristics associated with smoking in our sample included: area of residence, male gender, older age, White or Black race, being not married/partnered, or having Medicare/Medicaid. Factors associated with smoking in our sample are comparable to those associated with smoking in other FQHC populations. Reported rates of current smoking vary by FQHC setting but are consistently higher than the generally population (30% in one national FQHC sample, 26% in a sample of Ohio clinics).^12,13^

Previous studies have reported that current smokers experience more adverse health outcomes than former smokers and that smoking-related health risks decline after quitting.^3,17^ In our sample, former smokers had an equal or higher odds of many smoking-related health conditions as current smokers, although the temporal relationship between quitting smoking and disease development could not be established. The high burden of chronic disease among our patients who quit smoking may be due to many lower income individuals continuing to smoke until they become medically ill.^18,19^

A medical diagnosis has been identified as a critical teachable moment.^20^ Lindsay et al found a medical diagnosis to be the most common factor influencing a quit attempt (48%), followed by social factors (47%) and respiratory symptoms (36%).^20^ For patients with comorbid conditions, smoking affects treatment efficacy and promotes other adverse health outcomes.^21^ Increasing efforts to engage the patients in smoking cessation at earlier junctions is essential for low-income or disadvantaged populations with a high burden of comorbidities.

In the current study, patients reporting light and non-daily smoking had increased odds of several health conditions including asthma, COPD, emphysema, stroke, and peripheral vascular disease. These results add to a growing literature demonstrating that light and intermittent smoking carries health risks.^22^ Over the past several decades, the number of cigarettes smoked per day by active smokers has decreased significantly.^23^ Despite evidencing physiological indicators of addiction,^24,25^ individuals who smoke fewer cigarettes per day or are intermittent or non-daily smokers are less likely to consider themselves smokers. This increasingly common category of smokers is less likely to be identified as part of clinical practice.^26^ Clinical methods such as utilizing tobacco screening items designed to identify non-daily and light smoking in clinical practice should be implemented in FQHC clinics.

Our study replicates previous research findings that the odds of health conditions often differ by race or ethnicity. Black and White patients had the highest odds of lung cancer and chronic lung disease in our sample, and Hispanic people had the lowest. Choi et al reported on racial differences in health conditions from a sample of 8926 adults from the Health and Retirement Study.^27^ Similar to our sample, Black respondents had higher odds of hypertension, and Hispanic and Black people had higher odds of diabetes than non-Hispanic White people. The odds of cancer and lung disease were highest for White people. Assari et al reported differences in the prevalence of chronic lung disease by race/ethnicity.^28^ Black or Hispanic patients were at a decreased risk for chronic lung disease after controlling for other factors, although higher socioeconomic status was less protective for non-White people.

Some articles have found that health conditions for smokers differ by race or ethnicity. Haiman et al examined rates of smoking-related lung cancer by race and ethnicity and found Hispanic and Japanese American smokers to have the lowest risk and Black, White, and Native Hawaiian smokers to have the highest.^29^ The risks were moderated by the number of cigarettes smoked per day, with racial differences being the most pronounced with fewer cigarettes smoked per day. The racial differences were not significant when the number of cigarettes per day exceeded 30. In our analysis, the increase in emphysema and respiratory failure for smokers versus never smokers was higher for Black patients than for Hispanic patients. In addition, although Black patients had the highest odds of stroke and cardiovascular disease, the difference in the odds of these conditions for smokers compared with never smokers was significantly greater for White patients compared with Black or Hispanic patients. A possible explanation for this could be that other comorbid conditions and risk factors for stroke and cardiovascular disease such as high cholesterol, blood pressure, diabetes, obesity, and inactivity may more often exist for Black patients.

### Health equity implications

Our findings along with those in the extant literature underscore the continued and disproportionate levels of tobacco use and disease among low-income patients across different racial/ethnic groups. Smoking rates among our patient population were higher than national averages (14.0–16.7% nationally vs. 20.1% in our FQHC sample)^2^ and the general population in Chicago (13%).^30^ Rates of asthma and diabetes were also higher (14.8% and 17.4% in our sample vs. 9.4% and 12.0% for Chicago).^30^ Given that lower income individuals receive health care in FQHCs, this setting represents a viable option for reducing tobacco-related health disparities associated with socioeconomic status.

FQHCs should lead initiatives to obtain up-to-date information on tobacco use from patients and proactively reach out to tobacco users to offer smoking cessation advice, cessation medications, and referrals to cessation services as the desire to quit may change from month to month. Health system patient portals are a promising method for reaching out to patients to update their smoking status and offer smoking cessation services.^31,32^ In addition, patient navigation is a recognized and evidence-based approach for increasing patient access to health care and reducing health inequalities.^33–35^

### Limitations

This study used a cross-sectional survey design, and causation cannot be established. Data were from a single FQHC system in a large urban area. Findings may not generalize to other geographical locations. Information on smoking status, diagnoses, service use, and death was limited to what was documented in patients' health records and may have been inconsistently reported. Data on smoking were self-reported, and self-reported bias should be considered. Finally, data on duration of cigarette use in years and dose (average number of cigarettes per day) were unreliable, and the effect of these factors on health conditions could not be determined. In light of these limitations, additional studies are needed to replicate our study findings.

## Conclusions

Smoking rates were elevated among patients receiving care of an FQHC and were strongly associated with chronic health conditions and increased health care utilization. Along with heavy smokers, former smokers had the highest disease burden and health care utilization levels. In addition, the disease burden in our population was found to differ by race/ethnicity. Early intervention for smoking cessation remains a necessary priority for patients treated in safety net clinics. System-level innovations are needed to increase tobacco control efforts in FQHC settings to promote health equity.

## Data Availability

The data sets used and/or analyzed during the current study are available from the corresponding author upon reasonable request.

## Abbreviations Used

BMI: body mass index
CI: confidence interval
COPD: chronic obstructive pulmonary disease
CVD: cardiovascular disease
ED: emergency department
FQHC: federally qualified health center
IP: inpatient
IRR: incidence rate ratio
MSHC: Mile Square Health Center
OR: odds ratio
PVD: peripheral vascular disease
